# Anesthesia’s Influence on Postoperative In-Hospital Morbidity–Mortality in Proximal Femoral Fractures in the Elderly

**DOI:** 10.3390/medicina60091446

**Published:** 2024-09-04

**Authors:** Oded Hershkovich, Inga Tetroashvili, Adam Lee Goldstein, Raphael Lotan

**Affiliations:** 1 Department of Orthopedic Surgery, Wolfson Medical Center, Affiliated to the Sackler School of Medicine, Holon 5822012, Israel; dr.lotan@gmail.com; 2 Department of Anesthesia, Wolfson Medical Center, Affiliated to the Sackler School of Medicine, Holon 5822012, Israel; ingat@wmc.gov.il; 3 Trauma Unit, Wolfson Medical Center, Affiliated to the Sackler School of Medicine, Holon 5822012, Israel; adamg.barefoot@gmail.com

**Keywords:** mortality, femur, fracture, anesthesia

## Abstract

*Background and Objectives:* The incidence of proximal femoral fractures (PFFs) is rising, causing significant morbidity and mortality. Regional anesthesia (RA)’s benefits include the avoidance of intubation and mechanical ventilation, decreased blood loss, and improved analgesia. General anesthesia (GA) offers improved hemodynamic stability. This study examines the in-hospital post-surgical morbidity and mortality seen in PFFs in a cohort of the elderly undergoing GA or RA. *Materials and Methods:* This is a retrospective cohort study of 319 PFF patients older than 65 years over a single year. *Results:* In total, 73.7% of patients underwent GA. The patient characteristics were identical between groups, except for smoking. Hypertension was the most frequent comorbidity, followed by hyperlipidemia, NIDDM, and IHD. The overall patient complication rate was 11.4%. Pneumonia was the most common complication (5.1% in GA, 8.4% in RA). A total of 0.9% of patients required ICU admission. Overall, the in-hospital mortality rate was 2.3%, with no statistically significant difference between GA and RA. The GA and RA cohorts were similar in terms of their patient demographics, medical history, and preoperative parameters. In total, 73% of surgeries were under GA. No statistically significant differences were found in total anesthesia time or complication rates. *Conclusions:* We did not find a difference between general and spinal anesthesia regarding complication rates, anesthesia time, or morbidity. General anesthesia remains best suited for patients receiving anticoagulation treatment and undergoing semi-urgent surgery, but, other than that, the mode of anesthesia administered remains up to the anesthesiologist’s preference.

## 1. Introduction

The prevalence of proximal femoral fractures (PFFs) in the elderly population is rising, leading to significant morbidity and mortality in an already frail population [1,2]. Since 1990, when there were 1.66 million annual cases worldwide, there has been a steady rise toward the expected 6.26 million yearly cases by 2050, primarily driven by increased life expectancy [3]. The vast majority of these cases require surgical intervention [4], a process complicated by the advanced age and existing comorbidities in this patient population. These challenges are heightened due to the common presence of chronic diseases and age-related frailty, which significantly affect perioperative management and outcomes. 

Elderly patients with PFFs suffer considerable morbidity, mortality, and disability following surgery. In-hospital mortality rates hover around 5%, with about 10% of patients succumbing within the first month post-surgery due to complications primarily related to pulmonary and cardiovascular issues [5,6,7,8]. This high rate of adverse outcomes necessitates careful consideration of the anesthesia type used during surgical treatment. Regional anesthesia has gained attention for its potential benefits in the elderly undergoing PFF surgery. The avoidance of general anesthesia’s respiratory complications is particularly significant, given the high prevalence of chronic obstructive pulmonary disease (COPD) and other pulmonary conditions in this population. Furthermore, regional anesthesia techniques such as spinal anesthesia can offer superior postoperative pain management, which is crucial for early mobilization and rehabilitation, ultimately reducing the length of hospital stay and improving long-term outcomes. The use of regional anesthesia, including epidural, spinal, or peripheral nerve blocks, has been investigated as a strategy for mitigating the risk of postoperative complications [9,10]. Proposed mechanisms for the improved outcomes associated with regional anesthesia include the avoidance of intubation and mechanical ventilation, which can reduce the incidence of pulmonary complications, decrease intraoperative blood loss, and lead to better postoperative pain control [11,12].

On the other hand, general anesthesia, despite requiring intubation and the potential for mechanical ventilation, may offer better hemodynamic stability during surgery compared to regional anesthesia [11]. This is particularly relevant in elderly patients, who often have compromised cardiovascular systems and may not tolerate the hypotensive effects sometimes associated with regional anesthesia.

However, the choice between regional and general anesthesia is not straightforward. General anesthesia, while carrying the risk of pulmonary complications due to intubation, can provide more stable intraoperative conditions. This stability is critical in elderly patients who often have complex cardiovascular comorbidities. Hemodynamic fluctuations during surgery can lead to adverse outcomes, and general anesthesia might offer a more controlled environment to manage these fluctuations [11]. The decision-making process must, therefore, consider the individual patient’s health status, the presence of comorbidities, and the specific risks associated with each type of anesthesia.

Several studies have investigated the impact of anesthesia types on postoperative outcomes in elderly patients undergoing hip surgery. Berggren et al. (1987) conducted a study on 57 elderly patients with femoral neck fractures, finding that 44% experienced postoperative confusion, particularly associated with anticholinergic drug use and pre-existing cognitive impairments [13]. Davis et al. (1981) compared spinal and general anesthesia in a similar cohort, concluding that spinal anesthesia led to fewer complications, such as postoperative confusion and respiratory issues [14]. Juelsgaard et al. (1998) focused on perioperative myocardial ischemia and found that incremental spinal anesthesia reduced the incidence of myocardial events compared to single-dose spinal or general anesthesia [15]. Similarly, McKenzie et al. (1980) observed that spinal anesthesia resulted in better postoperative oxygenation and lower perioperative mortality rates than general anesthesia [16]. Racle et al. (1986) further supported these findings by demonstrating fewer cognitive and respiratory complications with spinal anesthesia in older women [17]. Neuman et al. (2012) conducted a comprehensive retrospective cohort study involving over 18,000 patients across 126 hospitals, revealing that regional anesthesia was associated with a lower risk of in-hospital mortality and fewer pulmonary and cardiovascular complications than general anesthesia. However, their study had limitations such as its retrospective design and the potential for selection bias, as the choice of anesthesia type was not randomized but based on clinical judgment and patient characteristics [18]. A more recent study by Neuman et al. (2021) on over 1600 participants found no significant difference in mortality or delirium between the anesthesia types but noted a lower risk of major complications with spinal anesthesia. The limitations of these findings include variability in study design, sample sizes, and potential confounding factors, such as patient comorbidities and surgical techniques, which necessitate further research to generalize these outcomes across broader populations [19].

In a more recent study, Turkmen et al. (2022) performed a retrospective analysis to assess the impact of anesthesia type on mortality rates in delayed femoral neck fracture surgeries [20]. Their findings suggested that the type of anesthesia used in hip surgeries performed 48 h to 5 days after trauma does not affect the 30-day mortality rate. Regardless of the type of anesthesia applied, patients died according to their comorbidities. However, the study’s retrospective nature and the potential confounding factors, such as the timing of the surgery and patient comorbidities, were noted as limitations [20]. Cao et al. (2023) conducted a systematic review and meta-analysis of randomized clinical trials comparing general and regional anesthesia in elderly patients undergoing hip fracture surgeries. The meta-analysis indicated that regional anesthesia was associated with intraoperative blood loss reduction and a shorter hospitalization period and duration of surgery. However, the two groups had no significant differences regarding the number of blood transfusions required, the duration of anesthesia, 30-day mortality, or postoperative delirium [21]. Liu et al. (2024) performed another systematic review and meta-analysis but found no significant difference in postoperative 30-day and 90-day mortality between regional and general anesthesia in hip fracture surgery patients, though regional anesthesia was associated with a lower incidence of intraoperative hypotension. In-hospital mortality and perioperative complications were not different between the two anesthesia techniques [22].

The increase in PFF cases is closely tied to demographic changes, notably the aging population. As life expectancy rises globally, the number of elderly individuals at risk for PFF also increases. This trend underscores the need for effective strategies to manage and treat PFF to reduce its associated morbidity and mortality. Surgical intervention remains the primary treatment modality for PFF, necessitating the exploration of perioperative management strategies that can optimize its outcome.

This study aims to contribute to this body of knowledge by analyzing a large cohort of elderly PFF patients undergoing surgery at a single center. By focusing on in-hospital morbidity and mortality rates, this study seeks to provide more precise data on the impact of anesthesia type on immediate postoperative outcomes. The single-center design ensures consistency in surgical and anesthetic protocols, potentially reducing some of the variability seen in multicenter studies.

## 2. Methods

This retrospective cohort study examined proximal femoral fractures (PFFs) in patients older than 65 over a calendar year. To ensure a focused analysis, several exclusion criteria were applied: patients on chronic anticoagulation treatment (Warfarin, Heparin), those with preoperative pulmonary emboli, patients deemed too sick to undergo surgery, individuals with a history of metastatic disease or multiple myeloma, multi-trauma patients, and those with paraplegia or quadriplegia. These criteria helped to create a more homogenous study population and reduce confounding variables.

The study involved a comprehensive review of patients’ medical records to gather demographic data, including age, gender, daily living activities (ADL), and whether the patients arrived from home or a nursing home. The medical history data collected encompassed conditions such as dementia, hypertension, hyperlipidemia, non-insulin-dependent diabetes mellitus (NIDDM), chronic lung disease, chronic renal failure (CRF), congestive heart failure (CHF), the presence of a pacemaker, and smoking status. Preoperative ASA scores were also recorded to assess the patient’s overall physical status and comorbidities before surgery.

The surgical data collected included the type of anesthesia used, the duration of the surgery, and the usage of blood products. Post-surgical morbidity was meticulously documented, focusing on complications such as pulmonary, cardiac, vascular, and renal issues, along with mortality rates. This comprehensive collection of data aimed to provide a clear picture of this patient population’s perioperative and postoperative outcomes.

A statistical analysis was conducted using IBM SPSS Statistics, version 25.0. Descriptive statistics were presented as means and standard deviations (M(SDs)) for continuous variables and as frequencies (*n*) with percentages (%) for categorical variables. Associations between patient characteristics and the type of anesthesia used were assessed using the Chi-square test for categorical variables and the t-test for continuous variables. Simple logistic regressions were employed to evaluate associations between the type of anesthesia used and postoperative parameters, with a *p*-value of less than 0.05 considered statistically significant. All reported *p*-values are two-tailed, ensuring a rigorous analysis of the data.

The study received approval from our institutional review committee, ensuring that all ethical guidelines were adhered to throughout the research process. This approval underscores our commitment to maintaining high ethical standards in clinical research and the protection of patient data. 

## 3. Results

The study cohort included 319 patients older than 65 admitted to the Orthopedic Surgery Department due to a proximal femoral fracture over one year. Patients underwent surgery under general anesthesia (235 cases; 73.7%) or spinal anesthesia (84 cases; 26.3%). General anesthesia was more common than spinal anesthesia, with 2.8 times more patients receiving this.

## 4. Patient Characteristics by Anesthesia

Table 1 presents the baseline characteristics of the patients, stratified by the type of anesthesia received. The mean age of the patients receiving general anesthesia was 83 ± 7.8 years, compared to 83.1 ± 7.9 years in the spinal anesthesia group, with no significant difference between the two (*p* = 0.915). The majority of patients in both groups arrived from home: 92.6% in the general anesthesia group and 90.4% in the spinal anesthesia group (*p* = 0.517). Their independence in the activities of daily living (ADL) was comparable between the groups, with 53.1% of general anesthesia patients and 56.0% of spinal anesthesia patients being independent in the ADL (*p* = 0.652).

The prevalence of dementia was slightly higher in the general anesthesia group (18.4%) compared to the spinal anesthesia group (16.7%), though this difference was not statistically significant (*p* = 0.726). The gender distribution was similar across both groups, with females constituting 70.7% of the general anesthesia group and 73.8% of the spinal anesthesia group (*p* = 0.584).

Patients’ ASA classification did not differ significantly between the two groups (*p* = 0.910), with the majority of patients classified as ASA 2 (57.4% in the general anesthesia group and 58.3% in the spinal anesthesia group). Chronic lung disease was present in 12.1% of general anesthesia patients and 11.9% of spinal anesthesia patients (*p* = 0.960). Hypertension was noted in 79.3% of the general anesthesia group and 82.1% of the spinal anesthesia group (*p* = 0.572), and hyperlipidemia was present in 60.9% and 56.0% of these groups, respectively (*p* = 0.419).

Smoking status revealed a statistically significant difference, with 2.7% of general anesthesia patients being smokers compared to 9.4% in the spinal anesthesia group (*p* = 0.003). The occurrence of cerebrovascular accidents (CVAs), ischemic heart disease (IHD), non-insulin-dependent diabetes mellitus (NIDDM), congestive heart failure (CHF), and chronic renal failure (CRF) showed no significant differences between the two groups (*p*-values of 0.663, 0.885, 0.307, 0.344, and 0.412, respectively).

Preoperative electrocardiogram (ECG) abnormalities were similar between the groups, at 6.6% in the general anesthesia group and 6.0% in the spinal anesthesia group (*p* = 0.824). Preoperative chest X-rays were largely normal in both groups (92.6% of general anesthesia and 95.2% of spinal anesthesia groups; *p* = 0.179).

Regarding surgical procedures, a proximal femoral nail (PFN) was the most common surgery performed, occurring in 56.2% of the general anesthesia group and 66.7% of the spinal anesthesia group, showing a significant difference (*p* < 0.001). Dynamic hip screw (DHS), Targon, and bipolar hemiarthroplasty (BPHA) surgeries were less common, with varying distributions between the groups.

The average surgery time was 54.6 ± 25.4 min for general anesthesia and 46 ± 18.4 min for spinal anesthesia (*p* = 0.854). The average anesthesia time was 85.8 ± 28.7 min for general anesthesia and 84.4 ± 30.7 min for spinal anesthesia (*p* = 0.909). Intraoperative complications were rare, occurring in 0.8% of general anesthesia patients and none of the spinal anesthesia patients (*p* = 0.417).

## 5. Postoperative Morbidity and Mortality by Anesthesia

Table 2 outlines the postoperative morbidity and mortality outcomes stratified by the type of anesthesia used. The incidence of pneumonia was higher in the spinal anesthesia group (8.4%) compared to the general anesthesia group (5.1%), although this difference was not statistically significant (*p* = 0.28). Pulmonary embolism occurred in 0.9% of general anesthesia patients and 1.2% of spinal anesthesia patients (*p* = 0.77).

The exacerbation of chronic obstructive pulmonary disease (COPD) was noted in 1.7% of general anesthesia patients and 3.6% of spinal anesthesia patients (*p* = 0.31). Atrial fibrillation occurred in 1.7% of the general anesthesia group, with no cases reported in the spinal anesthesia group (*p* = 0.23). Acute renal failure was slightly more common in the general anesthesia group (2.1%) compared to the spinal anesthesia group (1.2%) (*p* = 0.59).

The rates of ICU admission were low in both groups, at 0.9% in the general anesthesia group and 1.2% in the spinal anesthesia group (*p* = 0.77). Mortality was higher in the general anesthesia group (3%) compared to the spinal anesthesia group (1.2%), but this difference did not reach statistical significance (*p* = 0.37).

## 6. Discussion

Proximal femoral fractures in the frail elderly population carry significant morbidity and mortality risks. The ongoing debate regarding the role of anesthesia in mitigating their complication rate remains unresolved [18,19]. Orthopedic surgeons often prefer quick general anesthesia due to the perceived benefit of improved hemodynamic stability. In contrast, anesthesiologists advocate for spinal anesthesia to avoid intubation, mechanical ventilation, and a decrease blood loss and enhance postoperative analgesia [11]. This dichotomy underscores the complexity of perioperative management in this vulnerable patient cohort.

In our study, the cohorts receiving general and spinal anesthesia were comparable in terms of their patient demographics, medical history, preoperative chest X-ray findings, and blood work. Despite these similarities, the majority of surgeries were performed under general anesthesia, accounting for 73.7% of cases. This distribution likely reflects an anesthetist’s selection bias towards general anesthesia, even though patient characteristics were similar across both groups. A notable finding that correlates with this bias is the significantly higher percentage of smokers in the spinal anesthesia cohort (9.4% compared to 2.7% in the general anesthesia cohort, *p* = 0.003). This suggests a probable preference for avoiding mechanical ventilation in patients with a history of smoking.

Contrary to the common orthopedic belief that spinal anesthesia may result in prolonged anesthesia time, our study did not find a statistically significant difference in total anesthesia time between the two groups (*p* = 0.909). This finding is significant as it challenges the perception that spinal anesthesia is associated with longer procedural times. The absence of a difference in surgical time between the cohorts further supports this, indicating that the type of anesthesia used does not impact the duration of the surgery itself.

The overall patient complication rate in our study was 11.4%, with pneumonia being the most common postoperative complication. Pneumonia occurred in 5.1% of the general anesthesia group and 8.4% of the spinal anesthesia group (*p* = 0.28, Table 2). Other complications, such as pulmonary emboli, COPD exacerbation, atrial fibrillation, and acute renal failure, were less common and did not show statistically significant differences between the two anesthesia types (Table 2). These findings suggest that, contrary to prevailing beliefs, spinal anesthesia does not necessarily reduce pulmonary complications compared to general anesthesia.

Our study also found that around 0.9% of all patients required ICU admission postoperatively. The overall in-hospital mortality rate was 2.3%, with no statistically significant difference between patients receiving general anesthesia and those receiving spinal anesthesia. This is a critical observation as it indicates that neither anesthesia type significantly impacts in-hospital mortality rates in elderly patients undergoing surgery for PFF.

The comparable outcomes in terms of postoperative complications and mortality between general and spinal anesthesia suggest that the choice of anesthesia may be guided more by individual patient characteristics and clinical scenarios rather than a generalized preference for one type over the other. For instance, while spinal anesthesia might be preferred in patients with respiratory issues to avoid intubation, general anesthesia could be more suitable for patients where hemodynamic stability is a concern.

Our findings contribute to the ongoing discourse on the optimal anesthesia approach for elderly patients with PFF. By providing data from a single-center large-cohort study, we offer a more nuanced understanding of how anesthesia type impacts perioperative and postoperative outcomes. The lack of significant differences in key outcomes challenges some of the conventional beliefs held about anesthesia and underscores the need for individualized patient assessment and decision-making in the choice of anesthesia.

Despite the valuable insights gained from this study, several limitations must be acknowledged. Firstly, its retrospective design inherently limits our ability to control for all potential confounding variables, which could influence the observed outcomes. The study’s reliance on medical records for data collection introduces the risk of missing or inaccurately recorded information, which could impact the reliability of the results. Additionally, the single-center nature of the study may limit the generalizability of the findings to other settings or populations, as institutional practices and patient demographics can vary significantly. Selection bias is another notable limitation. The choice of anesthesia type was not randomized but rather based on the clinical judgment of the attending anesthesiologist, potentially introducing bias related to anesthesiologists’ preferences and patient characteristics not fully captured in the data. Although efforts were made to account for these differences, residual confounding cannot be entirely ruled out.

Furthermore, the study’s exclusion criteria, including patients on chronic anticoagulation treatment or those deemed too sick for surgery, may limit the applicability of the findings to the broader population of elderly patients with proximal femoral fractures. These criteria might exclude some of the most vulnerable patients, potentially underestimating the true complication rates and mortality associated with both anesthesia types.

The relatively short follow-up period, focusing primarily on in-hospital outcomes, does not provide information on longer-term complications, functional recovery, or quality of life post-discharge. Future studies with extended follow-up periods are needed to fully understand the long-term implications of anesthesia choice in this patient population.

Lastly, the substantial sample size of this study may still be insufficient to detect small but clinically meaningful differences in rare complications. Larger multicenter studies with randomized designs are warranted to validate these findings and provide more robust evidence to guide clinical practice. In summary, while this study makes important contributions to understanding the impact of anesthesia type on outcomes in elderly patients with proximal femoral fractures, its findings should be interpreted within the context of these limitations. Further research is needed to address these limitations and enhance the evidence base guiding anesthesia management in this high-risk population.

More extensive studies, especially randomized controlled trials, are needed, such as the study by Neuman et al. [19], who published results that are similar to our research.

## 7. Conclusions

In conclusion, while the type of anesthesia used in elderly patients with PFF does not significantly affect their overall complication rates or in-hospital mortality, the decision should still be tailored to the patient’s specific medical condition and risk factors. Further research with larger sample sizes and multicenter data would be valuable in confirming these findings and potentially refining guidelines for anesthesia management in this high-risk population. This approach aims to enhance perioperative care and improve the overall outcomes for elderly patients undergoing surgery for proximal femoral fractures.

## Figures and Tables

**Table 1:** Patients’ characteristics by anesthesia.

Variables	Anesthesia		*p*-Value
	General (%)	Spinal (%)	
Age ^ƾ^	83 ± 7.8	83.1 ± 7.9	0.915
Arriving from home	92.6	90.4	0.517
ADL independent	53.1	56.0	0.652
Dementia	18.4	16.7	0.726
Gender—female	70.7	73.8	0.584
ASA classification			0.910
1	4.7	3.6	
2	57.4	58.3	
3	37.9	38.1	
Chronic lung disease	12.1	11.9	0.960
Hypertension	79.3	82.1	0.572
Hyperlipidemia	60.9	56.0	0.419
Smoking	2.7	9.4	0.003 ^a^
CVA	12.5	10.7	0.663
IHD	24.2	25.0	0.885
NIDDM	32.0	38.1	0.307
CHF	7.8	4.8	0.344
CRF	10.2	7.1	0.412
Abnormal preoperative ECG	6.6	6.0	0.824
Preoperative X-ray			0.179
Normal	92.6	95.2	
Congestion	0.8	0	
Effusion	2.7	0	
Granuloma	1.2	0	
Emphysema	0.8	1.2	
Infiltration	2.0	3.6	
Surgery	*n* (%)	*n* (%)	<0.001
PFN	132 (56.2)	56 (66.7)	
DHS	31 (13.2)	11 (13.1)	
Targon	5 (2.1)	2 (2.4)	
BPHA	67 (28.5)	15 (17.9)	
Surgery time (min) ƾ	54.6 ± 25.4	46 ± 18.4	0.854
Anesthesia time (min)	85.8 ± 28.7	84.4 ± 30.7	0.909
Intraoperative complications	0.8	0	0.417
Postoperative complications			

^ƾ^ The age and time of surgery are given in terms of the mean (standard deviation). ^a^ *p*-value from Fisher exact test, as the chi-square assumption regarding the cells’ size was violated (two cells (50.0%) have an expected count of less than 5).

**Table 2:** Postoperative morbidity and mortality by anesthesia.

	General Anesthesia	Spinal Anesthesia	*p*-Value
Pneumonia	5.1	8.4	0.28
Pulmonary Embolism	0.9	1.2	0.77
COPD Exacerbation	1.7	3.6	0.31
Atrial Fibrillation	1.7	0	0.23
Acute Renal Failure	2.1	1.2	0.59
ICU Admission	0.9	1.2	0.77
Death	3	1.2	0.37

## Data Availability

The complete data are available under a confidentiality restriction.

## Abbreviations

Proximal femoral fractures = PFFs; regional anesthesia = RA; general anesthesia = GA; intensive care unit = ICU.
